# Primary angiosarcoma of the breast: a case report

**DOI:** 10.11604/pamj.2019.33.134.17414

**Published:** 2019-06-24

**Authors:** Mohamed Mouhoub, Achraf Miry, Anass Haloui, Nassira Karich, Imane Kamaoui, Saad Benkirane, Amal Bennani

**Affiliations:** 1 Department of Pathology, Mohamed VI University Hospital, Faculty of Medicine and Pharmacy of Oujda, Morocco; 2 Radiology Department, Mohamed VI university Hospital, Oujda, Morocco; 3 Gynecology Department, Mohamed VI University Hospital, Oujda, Morocco

**Keywords:** Primary angiosarcoma, breast, pathology, diagnosis

## Abstract

Primary angiosarcoma of the breast is an extremely rare tumour with a difficult diagnosis and poor prognosis. We report a case of primary breast angiosarcoma diagnosed in the pathology department of the University Hospital of Oujda. An analysis of the epidemiological, diagnostic and therapeutic aspects of this type of tumour is made in this manuscript. Mastectomy is the standard treatment; the place of radiotherapy and chemotherapy is not well established. We report a case of a 18- year-old woman having an infectious symptomatology of the right breast for which she received an anti-infectious therapy inducing regression of inflammatory symptoms presented with a quick growing mass. Initial core needle biopsy showed a malignant vascular proliferation. The patient underwent a mastectomy. The tumor histology showed papillary formations and vascular structures lined by atypical cells with hyperchromatic nucleus and eosinophilic cytoplasm. The tumor cells expressed CD34 and CD31 but were negative for cytokeratin. The diagnosis of angiosarcoma grade I was made. The patient is now receiving chemotherapy. She is still alive.

## Introduction

Angiosarcomas of the breast are very rare malignant tumours accounting for less than 0.05% [1] of all breast conjunctive vascular tumours. They are characterized by a high malignity and a fast growth rate [2] and evolve to rapid local recurrence and the appearance of visceral metastases. The diagnosis is essentially based on the histological assessment of the excised sample. The treatment is based on simple mastectomy. Our work is based on a case report with a review of the literature and aims to analyse the epidemiological, diagnostic and therapeutic aspects of this disease.

## Patient and observation

**Figure 1:**
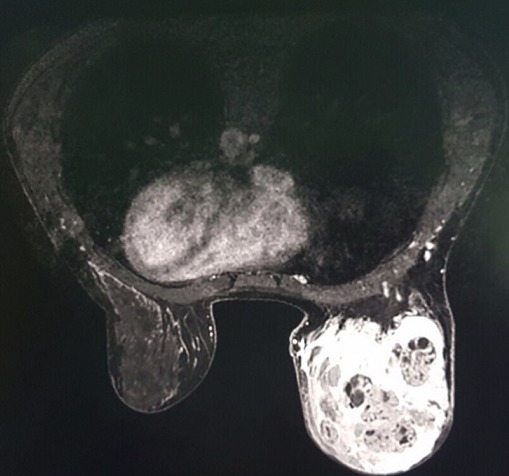
Right breast’s mass with solid and hyper vascularized components

**Figure 2:**
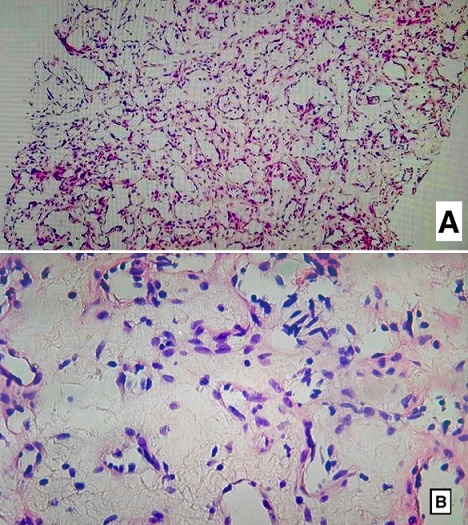
(A) microphotography showing the vascular nature of the malignant proliferation at low; (B) medium power view

**Figure 3:**
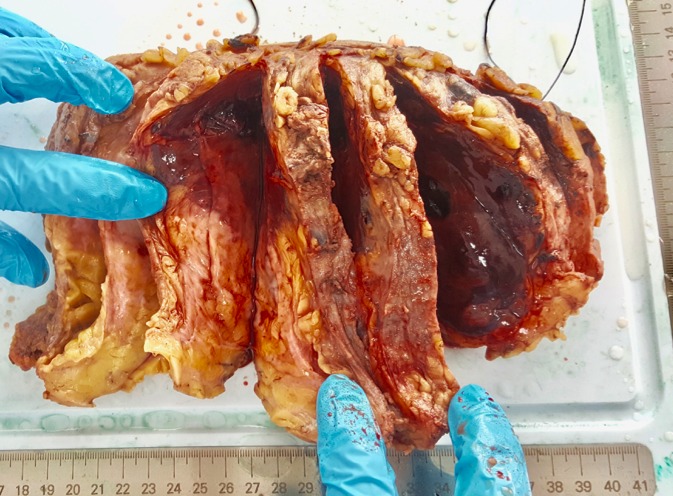
Gross examination revealing the presence of a bluish mass with haemorrhagic changes

**Figure 4:**
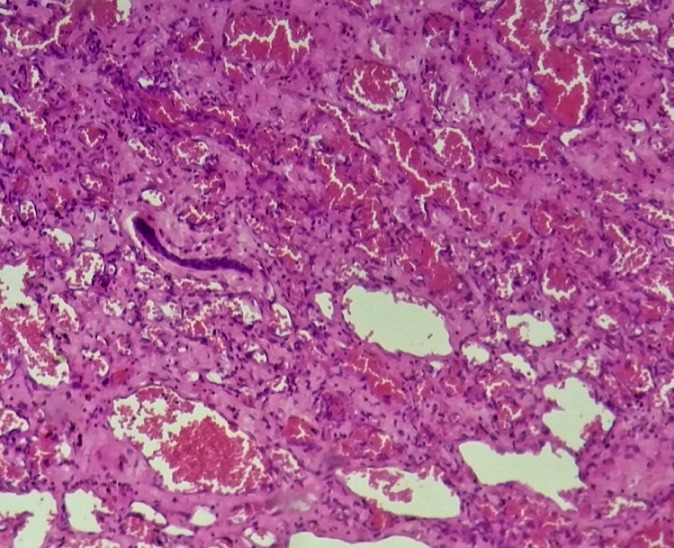
Microphotography showing the angiosarcomatous proliferation made of endothelial cells

**Figure 5:**
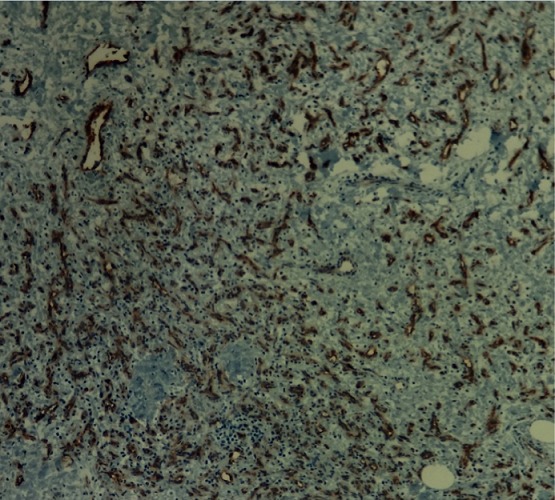
Microphotography showing intense positivity to anti-CD34 antibodies

An 18 year-old woman referred having an infectious symptomatology of the right breast 5 months ago for which she received an anti-infectious therapy inducing regression of inflammatory symptoms presented with a quick growing mass involving the two inferior quadrants of the right breast, but did not consult any doctor until it reached 10cm in its size. She had no history of breast trauma and no breast cancer in her family. An ultrasound of the right breast was performed and revealed a significant infiltration of the breast fat tissue with multiple intra-mammary lesions that may be related to the collection of infectious origin. Breast MRI showed a 12 x 10 x 6cm mass with solid and hyper vascularized components with significant enhancement (Figure 1). Microbiopsies revealed a malignant proliferation made of dilated, branching vascular structures with a high mitotic index reaching 10 mitoses per 10 high power fields evoking a well-differentiated angiosarcoma (Figure 2). The immunohistochemical study using anti-CD31 and anti-CD34 antibodies showed a diffuse and intense staining of the neoplastic cells. A mastectomy with lymph node dissection was performed. The gross examination showed the presence of a 12.5x11x6 cm bluish mass with haemorrhagic changes (Figure 3). Microscopic examination confirmed the angiosarcomatous proliferation made of endothelial cells focally producing papillary formations and projecting tufts into the lumen of the vessels (Figure 4). The tumour cells infiltrate the adipose tissue, where vascular lumens become inconspicuous. The immunohistochemical study confirmed the angiosarcomatous nature of proliferation since there was a diffuse and intense positivity to anti-CD34 and anti-CD31 antibodies (Figure 5). Lymph nodes invasion was absent. Chest CT scan and bone scintigraphy revealed no metastases.

## Discussion

Primary angiosarcoma represents 0.05% of all mammary tumours and 8% of breast sarcomas [1, 3]. It occurs especially in women having an age between 30 and 40 years. The median survival term is typically 24 months with a five-year recurrence-free survival rate of only 33% [4]. Prognostic factors are the histological type, grade and size of the tumour as well as the involvement of surgical section margins [5]. The factors involved in carcinogenesis are exposure to vinyl chloride, arsenic and thorostat, foreign body-induced chronic irritation and the notion of localized trauma [6]. The primary breast angiosarcoma must be differentiated from radiotherapy induced mammary angiosarcoma. Clinically, the lesion always measures more than 2cm and presents as a painless nodule. Zellek *et al* found that the size of the tumour is correlated with the 10-year recurrence-free survival rate and that the prognosis is particularly unfavourable when the tumour size is greater than 10cm [7]. The skin may look purplish, reddish or angiomatous. Lymph nodes involvement is very rare (0 to 5% of cases) [8]. The radiologic findings are not specific. Mammography can reveal a dense, often calcified, well circumscribed mass, with sometimes a focal asymmetry of density [9]. On ultrasound examination, it can appear as a heterogeneous mass with liquid areas related to necrotic and haemorrhagic changes [10]. It can also appear as a heterogeneous area of different echogenicity without any real mass [9]. The Doppler proves the hypervascular character of the tumour. MRI finds a heterogeneous low- and high-signal intensity mass respectively in T1 and T2 weighted sequences. Sometimes, T1-weighted MRI shows high-signal intensity areas corresponding to haemorrhagic zones or venous lakes [11]. The enhancement depends on the grade of the tumour.

Histologically, three grades have been correlated with prognosis by Donnel *et al* and Mérino *et al* [5]. Type I corresponds to a low-grade tumour with no massive necrosis, haemorrhage or papillary formation. Type II is an intermediate grade tumour without necrosis or haemorrhage. Type III is a high-grade tumour and has the least favourable prognosis. A tumour can include these three histological types. The differential diagnosis depends on the grade: sinusoidal haemangioma, angiolipoma, angiomatosis, benign lymphangiothelioma, pseudoangiomatous stromal hyperplasia and atypical vascular lesions could be suspected in front of a grade I tumour [5]. In types III, the differential diagnoses include sarcomatoid carcinoma or another type of high-grade sarcomas for which vascular markers (CD 34, CD 31) are not expressed. Generally, depending on their location, we distinguish: cutaneous angiosarcomas (of the scalp), deep soft tissue angiosarcomas and breast angiosarcomas. These tumors develop from endothelial cells. Recently, Itakura *et al* [12] have demonstrated the overexpression of vascular endothelial growth factor (VEGF-A) and VEGFR-1 in different subtypes of angiosarcomas. The standard treatment is total mastectomy [13, 14]. The assessment of the resection margins is fundamental and is the main predictor of local recurrence. Axillary lymph nodes dissection is useless except in cases of palpable lymph nodes since lymphatic spreading is rare [6]. Adjuvant chemotherapy is disappointing, but a benefit in terms of overall survival and recurrence-free survival has been found for high-grade angiosarcomas [15]. One of the therapeutic perspectives is targeted therapy, using anti-growth factor antibodies. The exploitation of VEGF-A and VEGF-C and its VEGF-R1 receptor, the main vascular growth factor, offers an interesting therapeutic target for inhibiting angiogenesis [16].

## Conclusion

Our case enabled us is to recall the characteristics of this rare tumour that can occur in a patient with no history of irradiation. Its prognosis is particularly severe and requires early diagnosis and rapid treatment. Its clinical, radiological or histological diagnosis is difficult and is rarely diagnosed before surgery. The treatment of choice is simple mastectomy. The role of radiotherapy and chemotherapy remains not fully established.

## Competing interests

The authors declare no competing interests.
